# Genetic Fingerprinting of Wheat and Its Progenitors by Mitochondrial Gene orf256

**DOI:** 10.3390/biom2020228

**Published:** 2012-04-13

**Authors:** Ahmed M. El-Shehawi, Abdelmeguid I. Fahmi, Samy M. Sayed, Mona M. Elseehy

**Affiliations:** 1 Department of Biotechnology, Faculty of Science, Taif University, Taif 21974, Saudi Arabia; Email: abdelmegidfahmi@yahoo.com (A.F.); samy_mahmoud@hotmail.com (S.M.S.); 2 Department of Genetics, Faculty of Agriculture-Chatby, University of Alexandria, Alexandria, Egypt; Email: monaandahmed@yahoo.com (M.M.E.); 3 Department of Economic Entomology and Pesticides, Faculty of Agriculture, Cairo University, Cairo, Egypt

**Keywords:** wheat, *Triticum*, *Aegilops*, orf256, fingerprinting, phylogenetic, molecular evolution

## Abstract

orf256 is a wheat mitochondrial gene associated with cytoplasmic male sterility (CMS) that has different organization in various species. This study exploited the orf256 gene as a mitochondrial DNA marker to study the genetic fingerprint of *Triticum* and *Aegilops* species. PCR followed by sequencing of common parts of the orf256 gene were employed to determine the fingerprint and molecular evolution of *Triticum* and *Aegilops* species. Although many primer pairs were used, two pairs of orf256 specific primers (5:-94/C: 482, 5:253/C: 482), amplified DNA fragments of 576 bp and 230 bp respectively in all species were tested. A common 500 bp of nine species of *Triticum* and *Aegilops* were aligned and showed consistent results with that obtained from other similar chloroplast or nuclear genes. Base alignment showed that there were various numbers of base substitutions in all species compared to *S. cereal* (Sc) (the outgroup species). Phylogenetic relationship revealed similar locations and proximity on phylogenetic trees established using plastid and nuclear genes. The results of this study open a good route to use unknown function genes of mitochondria in studying the molecular relationships and evolution of wheat and complex plant genomes.

## 1. Introduction

Hexaploid bread wheat (*Triticum aestivum*) is a member of Triticeae tribe, which also includes barley (*Hordeum vulgare*) and rye (*Secale cereale*) as well as other diploid and tetraploid wheats. Meiotic studies indicated that the general evolution of the Triticeae tribe has been defined by divergence at the diploid level from a common diploid ancestor and convergence at the polyploid level involving the diverged diploid genomes [1]. Cytological and molecular studies provided information on the identity of donors and the patterns of genome evolution of the *Triticum/Aegilops* species [2]. The *Triticum* and *Aegilops* genera contain 13 diploid and 18 polyploid species [3]. *T. monococcum* includes the cultivated form *T. monococcum ssp*. *monococcum* and the wild form, *T. boeoticum*. There are two tetraploid wheat species: *Triticum temopheevii* (AAGG genome) and *Triticum turgidum* (AABB genome). Finally, there are two hexaploid wheats: *Triticum zhukovskyi* (AAAAGG) and *T. aestivum* (AABBDD), including several subspecies [3,4].

*T. aestivum* is hexaploid with a genome constitution of AABBDD, and was formed about 8,000 years ago from hybridization between *T. turgidum* (AABB) and *A. tauschii* (DD) [5,6,7]. The A genome originated with *T. urartu* (AA), which is closely related to *T. monococcum* (AA). *A. speltoides*, *A. bicornis, A. searsii, and A. sharonensis* appear to have diverged from a common ancestor at about the same time [8]. The grass family (Poaceae) diverged about 50–80 mya into the subfamilies Pooideae (tribe Triticeae containing wheat, barley, rye, *Aegilops sp*.), Panicoideae (tribe Maydeae containing maize), and Bambusoides (tribe Oryzeae containing rice) [9,10,11]. Maize and sorghum diverged about 16.5 mya [12]; wheat and barley diverged about 10–15 mya [9], with wheat and rye diverging about 7 mya [13]. The cytoplasms of *T. aestivum*, *T. temopheevii*, and *T. turgidum* originate from an ancestor like *A. speltoides* [14].

It is suggested that the ancestor *Aegilops speltoides* species (S genome) was the donor of what became the B genome of the bread and durum wheats [15]. It is believed that *A. speltoides* is the B genome donor [16] and the maternal donor of polyploid wheats [17,18].

Nuclear genes have been used in molecular phylogenetic analysis. Sequence alignment of nuclear genes encoding plastid acetyl-CoA carboxylase (ACCase) and plastid 3-phosphoglycerate kinase (PGK) were used in molecular phylogenetic analysis of the *Triticum* and *Aegilops* species. This included A, D, and S diploids and A genome polyploids using a system based on sequences of large fragments [4,19,20]. On the other hand, receptor-like kinase, Lrk, genes were used to study hexaploid wheat evolution from its progenitors, yet the study showed high conservation in gene content and organization [21]. Therefore, molecular evolution studies over a narrow time window with highly conserved genes is not an advantage because changes in DNA sequence and rearrangements are minimal.

The chimeric open reading frame, orf256, is located upstream of coxI in fertile, cytoplasmic male sterile (CMS), and fertility restored (FR) mitochondria from Tt [22,23]. The 5' flanking sequence from −228 to −1 and the first 33 nucleotides of the coding sequence of the orf256 are identical to those of coxI of Ta, but the rest of the orf256 sequence is not related to that of coxI [22]. The orf256 sequence was detected in various species of wheat relatives and progenitors, but was expressed as RNA only in Tt and *Aegilops speltoides*.

Previous studies on orf256 showed some interesting features including (1) the close evolutionary history of *T. aestivum and T. temopheevii*, (2) the absence of orf256 in the mitochondrial DNA of *T. aestivum*, its presence in *T. temopheevii*, and the presence of a related sequence in rice, (3) the specific transcriptional and translational characteristics of orf256 depending on the source of the nucleus and the relationship to cytoplasmic male sterility, and (4) the lack of a known function for orf256. This gives a good opportunity to follow changes in its sequence, its location, its rearrangement, and its presence or absence in *Triticum* and *Aegilops* species. These molecular characteristics of orf256 suggest that this is a rapidly changing gene and make it a suitable molecular handle for evolutionary studies. In this study, orf256 was used as a molecular tool to establish a DNA fingerprint and phylogenetic relationship among *Triticum* and *Aegilops* species and their evolutionary changes.

## 2. Experimental Section

### 2.1. DNA Primer

Various specific primers (Table 1) were designed on the sequence of orf256 gene to cover different parts of the gene [24].

**Table 1:** Nucleotide sequence of primers that were used to detect the *orf256* sequence using PCR. Primers with bold face font gave common positive PCR results with all tested species.

Primer Name	Sequence 5' 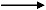 3'
**1.5: ** **−94**	**CCA TAT TCA CGC AAC TGA T**
2.5: −215	CTA CGA GAT CAC CTT CAC G
3.5: −190	CTG AGC CTT TAC GAG CAG G
4.5: 35	GCA GGT TTA CTG CTT TC
**5.5':253**	**CTGAGCCTTTACGAGCAGG**
6.C:785	TCA GAA TTA CTG AGC TAC
7.C:477	GGA ACG AAG CGC TTC ATC GA
8.C: 219	GCT TGG GGA TCC TGA ATC
**9.C:482**	**GAG ATG CTG TTT CCC ACA AC**
10.C:980	ATA GAG AGT CCC AAT ATC C
11.C:1469	GCT GTC ACT AGA ACG GAC C

### 2.2. Growth of Wheat Shoots

Wheat seeds were surface sterilized [13,25]. About 20 g of clean wheat seeds were soaked for 20 hr in 100 mL of 10 ppm ampicillin (Sigma) solution. The antibiotic solution was drained off and 100 mL of 0.1% silver nitrate (w/v) was added. Seeds were shaken vigorously for 10 min, and the silver nitrate solution was replaced with 100 mL of 0.5% of NaCl solution. After 10 min of vigorous shaking, seeds were rinsed three times with sterile, deionized water. Sterilized seeds were spread on 0.1% water agar in a sterilized plastic container and kept in the dark for 7–10 days at room temperature. Shoots were harvested and used directly for mitochondrial isolation or freeze dried for genomic DNA isolation.

### 2.3. Freeze drying of Wheat Shoots

10-day-old shoots were freeze dried in (Alpha 1–2 LO plus Christ, Vacuubrand, Germany). Dried shoots were ground in a coffee grinder to fine powder and used for DNA isolation.

### 2.4. Isolation of Mitochondria

Wheat mitochondria were isolated according to Song and Hedgcoth [26]. Mitochondrial pellets were stored at −20 °C.

### 2.5. Isolation of DNA

DNA was isolated from 10-day-old wheat shoots and or wheat mitochondria of *Triticum* and *Aegilops* species (Table 2). DNA was isolated using plant DNA isolation kit (Qiagen, California, USA) following manufacturer instructions. DNA concentration was estimated and used as PCR template. DNA samples were visualized on 1–2% agarose.

**Table 2:** *Triticum* and *Aegilops* species that were used in this study.

Species	Description	Ploidy level	Notes
*Triticum aestivum (Ta)*	Hexaploid wheat	AABBDD	
*Triticum timopheevii (Tt)*	wheat progenitor	GGAA	Possible source of B (G = B) genome
*Triticum turgidum (Ttu)*	wheat progenitor	BBAA	S = B
*Triticum monococcum monocuccom (Tm)*	wheat progenitor	AA	Source of A genome
*Triticum monocuccum bioeticum (Tb)*	wheat progenitor	AA	Source of A genome
*Aegilops speltoides (Asp)*	wheat progenitor	SS	S = B
*Aegilops bicornis (Ab)*	wheat progenitor	SS	
*Aegilops searsii (Ase)*	wheat progenitor	SS	
*Aegilops taushii (At)*	wheat progenitor	DD	Source of D genome
*Aegilops sharonensis (Ash)*	wheat progenitor	SS	
*Secale cereale (Sc)*	wheat relative		

### 2.6. PCR Amplification

Polymerase Chain Reaction (PCR) was used to amplify diagnostic fragments of orf256 using different combinations of primers. PCR was undertaken in 50 µL total volume containing 5 µL of 10X PCR buffer, 4 µL 25 mM MgCl2, 1 µL (10 ng) of DNA, 1 µL (100 ng, 125 picomole) of each primer (forward and reverse), 1 U of Taq DNA polymerase. PCR amplification conditions were initial denaturation at 95 °C for 5 min, denaturation at 95 °C for 1 min, annealing at 50 °C for 30 sec for 35 cycles, extension at 72 °C 1 min, and final extension at 72 °C for 5 min.

### 2.7. Sequencing of the PCR Amplified Fragments

The common PCR fragments obtained were amplified in all *Triticum* and *Aegilops* species, especially T. turgidum, and were purified and sequenced [24]. Ten samples were sequenced for each species to eliminate the heteroplasmy possibility of mitochondrial genomes.

### 2.8. Sequence Alignment and Phylogenetic Relationship

The obtained DNA sequences of the orf256 amplified fragments were aligned using CLUSTALW [27]. The phylogenetic relationship among *Triticum* and *Aegilops* species was established using PHYLIP program on the Pasteur Institute Server [28].

## 3. Results and Discussion

### 3.1. PCR

**Figure 1:**
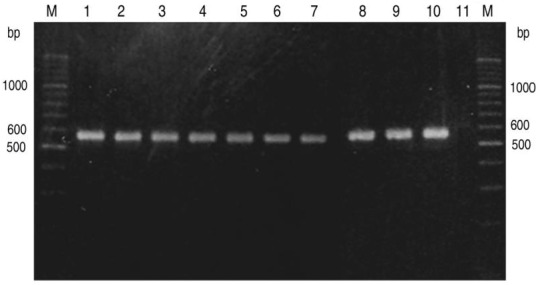
PCR product (576 bp) amplified using primer pair 5': −94 and C: 482. M: 100 bp DNA ladder;* 1: Tt, 2: Ttu, 3: Tm, 4: Tb, 5: Asp, 6: Ab, 7:Ase, 8: At, 9: Ash, 10:Sc, 11: Ta.* Full scientific names are shown in Table 2.

**Figure 2:**
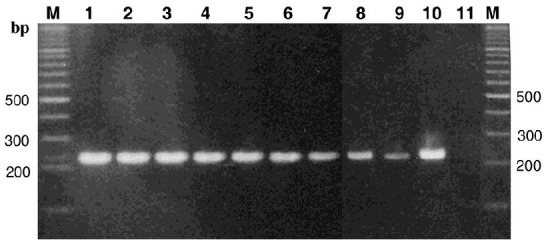
PCR product (230 bp) amplified using primer pair 5':253 and C: 482. *1: Tt, 2: Ttu, 3: Tm, 4: Tb, 5: Asp, 6: Ab, 7: Ase, 8: At, 9: Ash, 10:Sc, 11: Ta.* Full scientific names are shown in Table 2.

PCR was used to amplify DNA fragments from *Triticum* and *Aegilops* species. Using primer pair 5:-94/C: 482 (Table 1), PCR product of 576 bp was amplified (Figure 1) including *Triticum turgidum,* whereas using primer pair 5':253 and C: 482 resulted in the amplification of 230 bp fragment in all species tested (Figure 2). Other primer combinations (Table 1) amplified various fragments from different species except *Triticum turgidum*; therefore, we limited the comparison to these two fragments.

### 3.2. Sequencing and Bioinformatic Analysis of PCR Products

**Figure 3:**
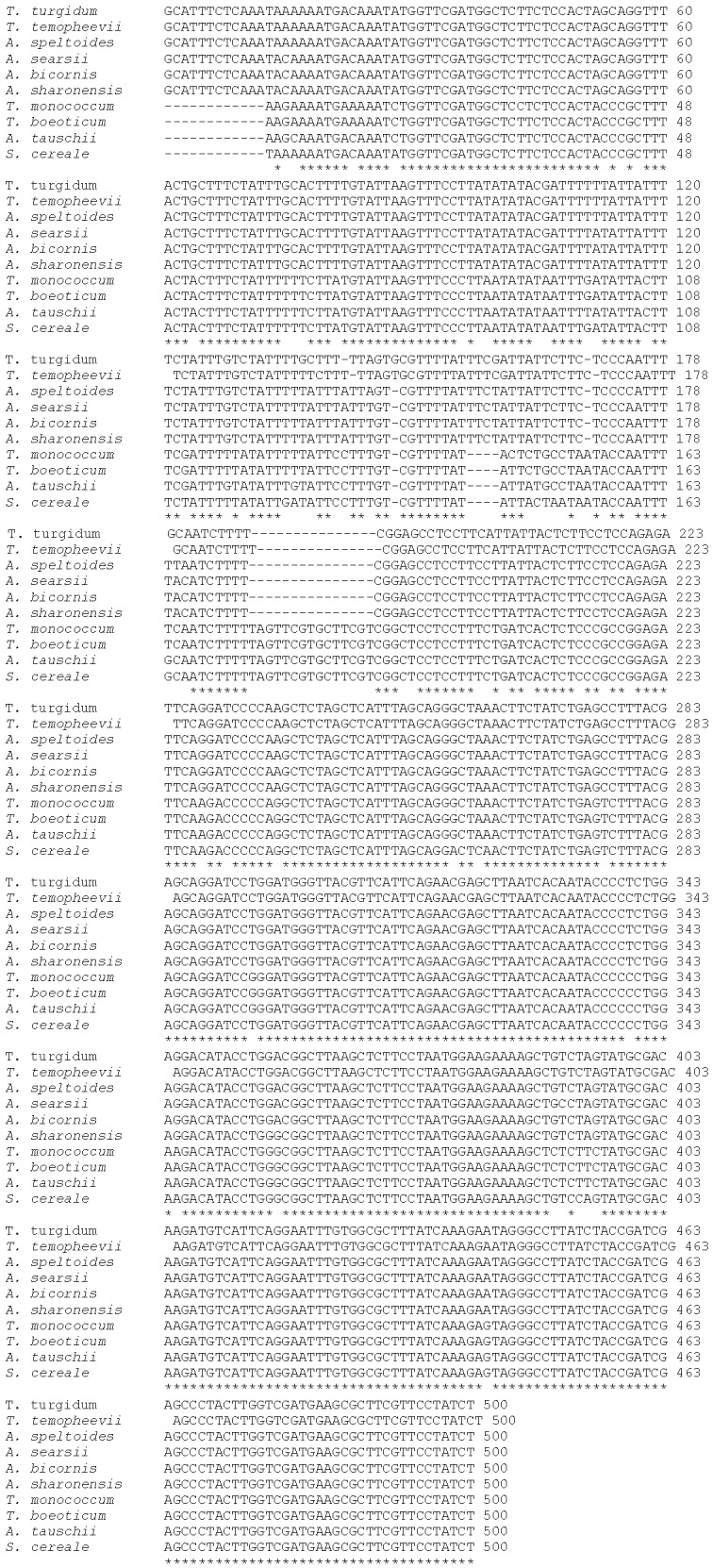
Multiple alignment of 500 bp of *orf256* of *Triticum* and *Aegilops* species.

The large PCR fragment (576 bp) obtained with primers 5': −94 and C:482 was cleaned and sequenced from the nine *Triticum* and *Aegilops* species. The nine DNA sequences obtained were used for multiple alignment using ClustalW2 (Figure 3). Multiple alignments revealed many differences among the nine sequences used in this study. Generally, the 5' third of the aligned sequences showed the most drastic and significant differences, including cluster of deletions or single deletions in some species as well as base substitutions. The middle part of the sequence has fewer changes, whereas the 3' third is more conserved among the species under study. Alignment of 500 bp showed various numbers of base substitutions compared to *Secale cereale* (out of group species) (Table 3). It showed 49 base substitutions in *T. temopheevii* and *T. turgidum*; 50 base substitutions in *T. monococcum*; 47 base substitutions in *T. boeoticum*, *A. speltoides*, *A. bicornis*; 25 base substitutions in *A. searsii*; 22 base substitutions in *A. tauschii*; and 21 base substitutions in *A. sharonensis* (Table 3).

**Table 3:** Summary of PCR product size obtained and the number of base substitutions in *Triticum* and *Aegilops* species used in this study compared to *S. cereal* sequence.

Sequence obtained		Species									
		Ttu	Tt	Ase	Ab	Ash	Asp	Tm	Tb	At	Sc
Primers	5: −94/C:482	576	576	576	576	576	576	576	576	576	576
	5:253/C:482	230	230	230	230	230	230	230	230	230	230
Number of base substitutions		49	49	50	47	47	47	25	22	21	-

### 3.3. Phylogenetic Analysis

The longest orf256 sequence obtained from *T. turgidum* is 576 bp using primer pairs 5: −94/C: 482. Only 500 bp were used (76 bp were eliminated) because of gaps to establish a consensus phylogenetic tree. The tree was established using PHYLIP software on the Pasteur Institute website [28]. The consensus tree was calculated by the UPGMA method. Bootstrap values were calculated as percentages of 1000 trials. *Secale cereale* was used as outgroup species. Six data sets were included in the calculation of the consensus tree using the nine species. Set one included species *A. speltoides*, *A. sharonesis*, *A. bicornis*. *A. searsii*, *T. temopheevii*, *T. turgidum*. Set two included species *A. tauschii*, *T. boeoticum*, and* T. monococcum.* Set three included species *T. temopheevii* and *T. turgidum*. Set four included species *T. boeoticum* and *T. monococcum*. Set five included species *A. speltoides*, *A. sharonesis*, *A. bicornis*, *A. searsii.* Set six included species *A. sharonesis*, *A. bicornis*, and *A. searsii*.

**Figure 4:**
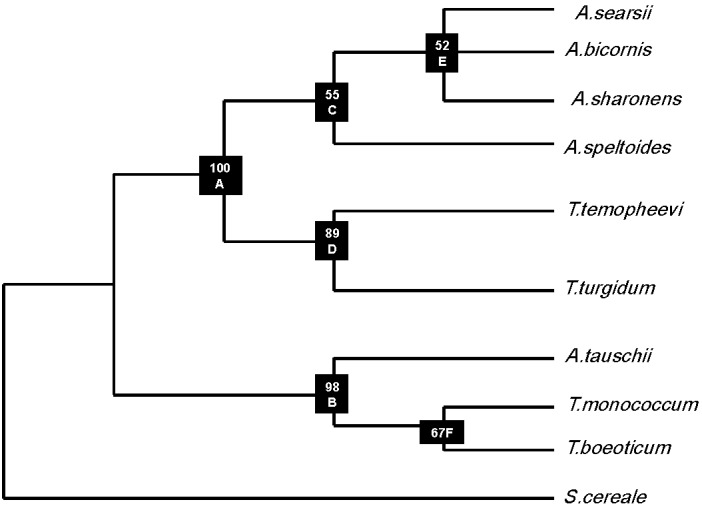
Consensus phylogenetic tree of *Triticum* and *Aegilops* species based on the common 500 bp of orf256 sequence and one thousand trials. Bootstraps (the numbers on the branches) indicate the number of times the partition of the species into the two sets, which are separated by that branch, occurred among the trees, out of 999.99 trees.

The consensus tree was established by making one thousand trials (Figure 4). The tree has two clades, A and B. Clade A which has the same location (bootstrap) in one thousand trials contains two branches, C and D. Branch C contains one species; *A. speltoides* and sub-branch E which contains three species *A. searsii*, *A. bicornis*, *A. sharonesis*. Branch D contains two species; *T. temopheevii* and *T. turgidum*. Clade B that has the same location (bootstrap) in 975 trials has one species; *A. tauschii* and one sub-branch F which included two species; *T. monococcum*, *T. boeoticum* (Figure 4).

### 3.4. Distance Matrix

DNA distances among studied species were calculated using DNAbars software on the Pasteur Institute website [28,Table 4]. *T. temopheevii* and *T. turgidum* were the closest species with DNA distance about 0.2. Also, minimum distances occurred between *A. serseaii* and *A. bicornis*, *A. bicornis* and *A. sharonesis, T. monococcum* and *T. boeoticum* with distances of 0.2, 0.2, and 0.4 respectively. The highest distance was between *T. temopheevii* and *A. speltoides* to *A. tauschii* with DNA distance of 33.78. *S. cereale* is the outgroup species. *T. temopheevii* and *A. speltoides* were the most separated species of *S. cereal* with DNA distance of 32.54, although they were located on different sub-branches of the consensus phylogenetic tree, whereas *T. boeoticum* and *A. taushii* were the closest species to *S. cereale* with DNA distance of 4.11, although they were located on different sub-branches of the consensus phylogenetic tree.

**Table 4:** DNA distances among *Triticum* and *Aegilops* species (1000 trials). Distance Matrix was calculated using the Jukes-Cantor correction method. Base positions 123 in the codon and gap weighting 0.0 were used.

Species	Ttu	Tt	Ase	Ab	Ash	Asp	Tm	Tb	At	Sc
*T.turgidum*	0.00									
*T.temopheevii*	**0.20**	0.00								
*A.searsii*	3.06	2.85	0.00							
*A.bicornis*	2.85	2.65	**0.20**	0.00						
*A.sharonensis*	3.06	2.85	0.40	0.20	0.00					
*A.speltoides*	2.44	2.23	1.41	1.21	1.41	0.00				
*T.monococcum*	32.85	33.16	33.16	32.85	32.54	33.16	0.00			
*T.boeoticum*	32.85	33.16	32.85	32.54	32.23	33.16	**0.40**	0.00		
*A.tauschii*	33.47	**33.78**	33.47	33.16	32.85	**33.78**	1.82	1.41	0.00	
*S.cereale*	32.23	**32.54**	32.23	31.93	31.62	**32.54**	4.53	**4.11**	**4.11**	0.00

*T. turgidum* gave negative results with other primer pair combinations (Table 1). Results previously obtained from other studies also suggested that this species has a partial orf256 sequence [13]. Common 500 bp were used to study the similarity among the nine different *Triticum* and *Aegilops* species. The phylogenetic relationship among studied species, although different, was still consistent with results obtained from previous studies which used other plastid and nuclear genes. In a study using 3 phosphoglycerate kinase (pgk-1) gene of *Triticum* and *Aegilops,* results revealed that some species showed similarity of location on the phylogenetic tree. *T. temopheevii* and *T. turgidum* along with *T. aestivum* (not included in this study since it does not have orf256 in its mitochondria DNA) showed closer location on the phylogenetic tree using acetyl-coA carboxylase (ACC-1) and 3-phosphoglycerate kinase (PGK-1) [4,29]. They were mapped on one sub-branch (sub-branch D, Figure 4). *A. speltoides* showed independent location from other *Aegilops* or *Triticum* species using the same genes (PGK-1) [4,29]. The present study showed a similar pattern because it is positioned on a separate branch with bootstrap of 55 (Figure 4). *A. searsii*, *A. bicornis*, and *A. sharonesis* showed a closer location in the present study. They were located on sub-branch E with bootstrap 52 (Figure 4). They showed similar relatedness [4] using 3-phosphoglycerate kinase (PGK-1) while they did not show this close proximity on the phylogenetic tree using acetyl-coA carboxylase (ACC-1) and 3-phosphoglycerate kinase genes (PGK-1) [29]. *Triticum monococcum* and *T. boeoticum* also showed close proximity location on phylogenetic trees using 3-phosphoglycerate kinase (PGK-1) [29], yet they did not show this close proximity on phylogenetic trees using the same gene ((PGK-1) [4]. *T. taushii* is located on an independent branch in this study (Figure 4), but in other studies, it showed close proximity with *T. aestivum* using ACC-1 and PGK-1 genes [4,29]. A partial sequence of *WAG-2* gene was used to study the molecular evolution of wheat and its relatives. Marked variations were reported in single nucleotide polymorphisms (SNIPS) and indel numbers. Similar topology of phylogenetic trees using *WGA-2* gene and the orf256 genes were obtained. For example, *A. tauchii* was located on one separate clade (clade III) using the *WGA-2* gene. A similar location on the phylogenetic tree was obtained using orf256 gene (30). *A. speltoides* and *T. turgidum* had close phylogenetic topology on trees established using *WGA-2* and orf256 genes. This supports the established idea that *A. taushii* (DD) is the source of D genome of *Triticum aestivum* (AABBDD).

From the data obtained from this study and similarities of our results with results obtained using other nuclear and plastid genes, it can be concluded that the orf256 represents a suitable molecular tool to study the relationship among *Triticum* and *Aegilops* species. Also, this introduces one more mitochondrial gene to study bioinformatic relationships among species with complex genomes which could lead to resolving their evolution at the molecular level. Orf256 and other genes could be used in monitoring gene transfer among cellular organelles, especially the nucleus and mitochondria, and tracking their evolutionary changes.

## 4. Conclusions

Wheat mitochondrial gene orf256 was used to study the phylogenetic and the evolutionary relationship among *Triticum* and *Aegilops* species. The results obtained were consistent with those obtained using plastid and nuclear genes. Also, the phylogenetic tree obtained from this study gave similar locations to many *Triticum* and *Aegilops* species which used plastid and nuclear genes. Data conclude that the orf256 gene of wheat mitochondrial DNA is a good molecular tool to study bioinformatic analysis of *Triticum* and *Aegilops* genomes.
